# Regressionsanalyse zur Berechnung des ROSC-Zeitpunkts – eine Machbarkeitsstudie

**DOI:** 10.1007/s00101-026-01648-4

**Published:** 2026-01-30

**Authors:** André Luckscheiter, W. Zink, M. Thiel, V. Schneider-Lindner

**Affiliations:** 1 Klinik für Anästhesie, Intensiv- und Schmerzmedizin/OP-Abteilung, BG Klinik Ludwigshafen, Ludwig-Gutmann-Str. 13, 67071 Ludwigshafen, Deutschland; 2 Medizinische Fakultät Mannheim, Universität Heidelberg, Mannheim, Deutschland; 3 Klinik für Anästhesiologie, Operative Intensivmedizin und Notfallmedizin, Klinikum Ludwigshafen, Ludwigshafen, Deutschland; 4 Klinik für Anästhesiologie und Intensivmedizin, Universitätsklinikum Mannheim, Mannheim, Deutschland

**Keywords:** Reanimation, Maschinelles Lernen, Maschinelle Vorhersagemodelle, Entscheidungsbäume, Statistische Modelle*, Resuscitation, Machine learning, Prediction methods, machine, Decision trees, Models, statistical*

## Abstract

**Hintergrund:**

Ein Regressionsmodell, welches die Dauer vom Beginn der Reanimation bis zum Einsetzen eines Spontankreislaufs (ROSC) abschätzt, könnte sowohl die Reanimationsversorgung als auch die Qualitätskontrolle in Registern verbessern. In dieser Studie sollen erstmals dessen Vorhersagegenauigkeit und Probleme für die weitergehende Modellentwicklung evaluiert werden.

**Methodik:**

Regressionsmodelle, basierend auf den M5P- sowie Random-Forest(RF)-Algorithmen sowie einer mittels M5P-modifizierten linearen Regression (LR), wurden an einer belgischen Kohorte aus 84 Personen mit ROSC erstellt und mittels Gütekriterien wie dem Korrelationskoeffizient (KK), Bestimmtheitsmaß R^2^ und der Wurzel der mittleren Fehlerquadratsumme (RMSE) in einem Kreuzvalidierungsverfahren bewertet.

**Ergebnisse:**

In der Kohorte waren 61,9 % männlich bei einem Durchschnittsalter von 65,7 Jahren. Ein defibrillierbarer Rhythmus lag in 27,7 % vor (Laienreanimationsquote 48,2 %). Die No-Flow-Time betrug 5,13 min sowie die Zeiten von Reanimationsbeginn zur ersten Defibrillation 7,81 min resp. bis zur Erstmedikation 11,31 min. Ein ROSC trat im Mittel 16,8 min nach Beginn der Reanimation ein. Den höchsten KK zeigte LR (0,73 [95 %-Konfidenzintervall, 95 %-KI 0,72–0,74]), R^2^ 0,53 [0,52–0,55] bei niedrigstem RMSE (6,76 min [6,63–6,90]). Nicht-signifikant unterschiedliche Werte erzielte M5P (KK 0,72 [0,70–0,73], R^2^ 0,52 [0,50–0,53], RMSE 6,84 min [6,69–6,99], *p* > 0,05). Dagegen schnitt RF signifikant schlechter ab (KK 0,62 [0,61–0,63], R^2^ 0,38 [0,37–0,40], RMSE 7,89 min [7,82–7,96], alle *p* < 0,01). Einzig für LR waren Mittelwert (*p* = 0,75) und Varianz (*p* = 0,15) statistisch nicht unterschiedlich von den Istwerten. Der Anteil an potenziell vorzeitig beendeten Reanimationen, bei denen der tatsächliche ROSC erst nach dem Zeitraum Regressionswert plus RMSE auftrat, reichte von 13 % (M5P) bis 18 % (LR).

**Schlussfolgerung:**

Die Zeitdauer vom Beginn der Reanimation bis zum ROSC ist potenziell abschätzbar. In dieser frühen Entwicklungsphase waren die individuellen Vorhersagen der Regressionsmodelle noch nicht ausreichend valide, möglicherweise aufgrund einer zu simplen Datenstruktur und zu geringer Kohortengröße. Jedoch zeigten sich Hinweise auf eine Anwendung zur Qualitätskontrolle im Sinne einer Analyse von tatsächlicher vs. beobachteter Zeitdauer. Zur Steigerung der Robustheit sollten die Ergebnisse daher anschließend an einer größeren Kohorte mit erweiterter Datenbasis auf Grundlage der Utstein-Kriterien evaluiert werden.

**Graphic abstract:**

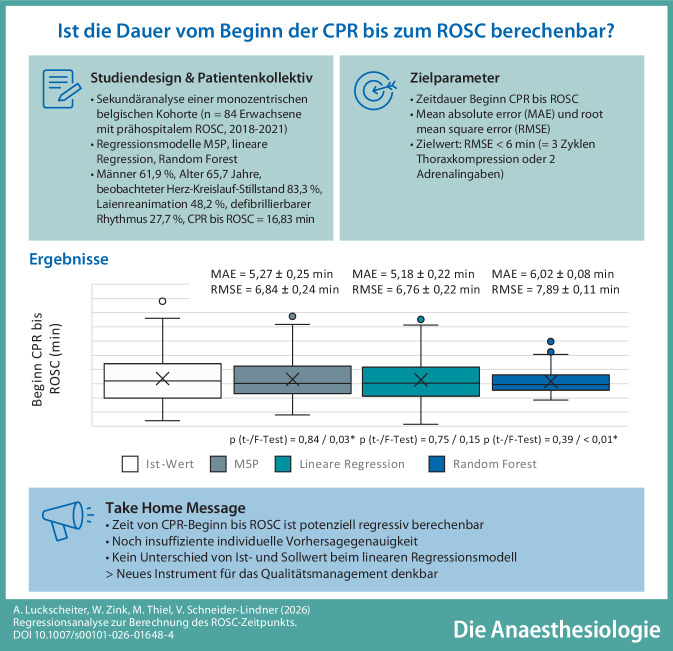

## Abstract (en)

**Background:**

A regression model to estimate the duration from the onset of resuscitation efforts to the return of spontaneous circulation (ROSC) could help improving both resuscitation care and the quality control of registries. This study aims to evaluate the prediction accuracy and to identify challenges for future model development.

**Method:**

Regression models based on M5P, random forest (RF) algorithms and a linear regression (LR) modified using M5P were retrospectively developed using a Belgian cohort of 84 individuals in whom ROSC was achieved. Model performance was assessed using quality metrics, such as the correlation coefficient (CC), coefficient of determination (R^2^), and root mean square error (RMSE) in a cross-validation approach.

**Results:**

In the cohort 61.9% were male with a mean age of 65.7 years. A shockable rhythm was present in 27.7% of cases and the bystander cardiopulmonary resuscitation (CPR) rate was 48.2%. The no-flow time averaged 5.13 min. The mean time from CPR onset to first defibrillation was 7.81 min and to first medication administration 11.31 min. The ROSC occurred after an average of 16.8 min, the LR showed the highest correlation (0.73, 95% confidence interval, CI 0.72–0.74) and R^2^ (0.53 [0.52–0.55]) along with the lowest RMSE (6.76 min [6.63–6.90]). The M5P yielded similar not significantly different values (CC 0.72 [0.70–0.73], R^2^ 0.52 [0.50–0.53], RMSE 6.84 min [6.69–6.99]). In contrast, RF performed significantly worse (CC 0.62 [0.61–0.63], R^2^ 0.38 [0.37–0.40], RMSE 7.89 min [7.82–7.96], all *p* < 0.01). Only LR showed no significant difference between predicted and actual values in terms of mean (*p* = 0.75) and variance (*p* = 0.15). The proportion of potentially prematurely terminated resuscitation attempts, defined as cases with actual ROSC occurring later than predicted ROSC plus RMSE, ranged from 13% (M5P) to 18% (LR).

**Conclusion:**

The duration from the start of CPR to ROSC appears to be a process that is suitable for modelling with machine learning algorithms. At this early stage of development, the individual regression models did not demonstrate sufficient validity possibly due to low sample size and simplified data structure; however, the findings indicated potential for an application as a quality assurance tool to compare actual vs. predicted time to ROSC. Therefore, to increase the robustness the results require further evaluation in a larger cohort with additional variables and improved data quality based on the Utstein criteria.

## Hinführung

Das Wiedereinsetzen eines Spontankreislaufs bei der präklinischen Reanimation unterliegt einer Wahrscheinlichkeit, die bedingt ist durch Patientencharakteristika, die Reanimationsumstände, das Ineinandergreifen der Rettungskettenglieder und qualitativ hochwertige Wiederbelebungsmaßnahmen. Die Studie möchte der Frage nachgehen, ob sich eine standardisierte Beschreibung des Reanimationsablaufs dahingehend nutzen lässt, mittels maschinellen Lernens die Zeitdauer vom Beginn der Reanimationsmaßnahmen bis zum ersten Spontankreislauf abzuschätzen, und welche Einsatzmöglichkeiten sich aus solch einem Modell ergeben würden.

## Einleitung

Jährlich ereignen sich in Deutschland ca. 150 präklinische Reanimationen/100.000 Einwohner, wobei in gut 40 % ein wiedereinsetzender Spontankreislauf (ROSC) erzielt werden kann [11]. In den letzten Jahrzehnten wurden neben präexistenten Faktoren wie Patientenalter und Vorerkrankungen auch einige prädisponierende Parameter identifiziert, welche die Wahrscheinlichkeit für einen ROSC erhöhen resp. mit einem guten neurologischen Outcome korrelieren. Faktoren wie Laienreanimation, optimierte Rettungsketten, Frühdefibrillation, verbessertes Atemwegsmanagement und hohe Qualität der Reanimationsmaßnahmen sind nicht nur Gegenstand der Leitlinienempfehlungen, sondern werden auch international einheitlich anhand der Utstein-Kriterien erfasst [1, 10, 23, 25, 28]. Dennoch sehen sich Notärzte während einer Reanimation immer wieder mit dem Problem des „resuscitation time bias“ konfrontiert [8]. Hierbei werden zwar mit steigender Dauer der Reanimationstherapie in Summe mehr ROSC erzielt, die Wahrscheinlichkeit für ein schlechtes neurologisches Outcome steigt allerdings ebenfalls stark an. Daher ist die Abschätzung der optimalen Reanimationsdauer Gegenstand aktueller Studien und von hoher klinischer Relevanz [8, 24]. So konnten Grunau et al. zeigen, dass nach 48 min bei initial defibrillierbarem und 15 min bei nichtdefibrillierbarem Initialrhythmus die Überlebenswahrscheinlichkeit unter 1 % sinkt [15]. Die Leitlinie empfiehlt die Einstellung der Wiederbelebungsversuche nur bei offensichtlich tödlichen Verletzungen bzw. der dokumentierten definitiven Ablehnung einer solchen Behandlung. Bei einer anhaltenden Asystolie über 20 min oder permanent niedrigen endtidalen CO_2_-Werten soll dagegen ein Abbruch der Maßnahmen lediglich erwogen werden [25]. Bei der Diskussion um eine mögliche Einstellung von Reanimationsmaßnahmen an der Einsatzstelle kommt erschwerend hinzu, dass die Möglichkeiten der Diagnostik und Therapie mit Blick auf die Ausstattung der Rettungsmittel gerade bei reversiblen Kreislaufursachen begrenzt sind. Daher besteht oftmals präklinisch noch immer eine gewisse Unsicherheit, ob und wann noch ein ROSC erzielt werden könnte. Maschinelles Lernen (ML) in der Reanimatologie stellt einen vielversprechenden Ansatz dar, Notärzte bei dieser Thematik rund um dem „resuscitation time bias“ zu unterstützen. Bisher existieren v. a. Modellierungen zur Wahrscheinlichkeit eines ROSC ohne zeitliche Auflösung zum Reanimation-Outcome [5, 6, 17, 19, 29]. Aktuelle Modelle zur ROSC-Wahrscheinlichkeit aus asiatischen Kohorten erzielten Sensitivitäten zwischen 0,71 und 0,84 und positiv-prädiktive Werte von 0,19–0,62 [2, 18, 26]. Einige wenige Untersuchungen zur zeitlichen Auflösung der ROSC-Wahrscheinlichkeit mittels ML legen nahe, dass die Ereigniswahrscheinlichkeit um Minute 10 am höchsten ist, bis Minute 20 annährend linear abfällt und sich dann asymptotisch dem Nullpunkt nähert. Ab Minute 30 nach Beginn der Reanimationsmaßnahmen steigt die kumulative Wahrscheinlichkeit für einen ROSC kaum noch an [24]. Ebenso hat sich die initiale ROSC-Wahrscheinlichkeit zwischen Minute 20 und 30 mindestens halbiert [8, 26]. Obgleich die Studien hinweisend auf zeitabhängige Einflussfaktoren für einen ROSC sind, wurde nicht versucht, den genauen Zeitpunkt des ROSC zu berechnen. Bei solch einer Regressionsanalyse wird mittels ML nicht die Ereigniswahrscheinlichkeit berechnet, sondern die Zeit bis zu dessen Eintreten. Mit Blick auf den „resuscitation time bias“ wäre diese Berechnung von hoher klinischer Relevanz, sei es zur Planung der Einsatztaktik, Weiterversorgung oder eben Abschätzung der neurologischen Prognose. Ziel der vorliegenden Studie war es daher, erstmals Regressionsmodelle zur Abschätzung der Zeitdauer bis zum ROSC ab Beginn der Reanimationsmaßnahmen zu erstellen und zu evaluieren. Es wurde die primäre Hypothese aufgestellt, dass die Fehlergenauigkeit, definiert als Wurzel der mittleren Fehlerquadratsumme („root mean square error“, RMSE) unter 6 min liegt (3 Zyklen Thoraxkompression bzw. 2 Adrenalingaben). Als sekundäre Hypothese wurde angenommen, dass sich die berechneten Mittelwerte und Varianzen nicht signifikant von den Istwerten unterscheiden. Aus den Ergebnissen sollen zudem Implikationen für die weitere Modellentwicklung und deren Einsatzmöglichkeiten abgeleitet werden.

## Methodik

Die vorliegende Studie wird nach dem Prinzip des „Transparent Reporting of a Multivariable Prediction Model for individual Prognosis or Diagnosis“ (TRIPOD) wiedergegeben [9]. Die Untersuchung ist eine Sekundärauswertung einer Studie von Malinverni et al. aus der Region Brüssel, Belgien [21]. Darin untersuchten die Autoren prospektiv an 92 Patienten mit präklinischen ROSC im Zeitraum November 2018 bis August 2021, ob die Titration von Sauerstoff nach dem Oxygen Reserve Index und der Sauerstoffsättigung oder anhand einzig der Sauerstoffsättigung zur Vermeidung einer Hyperoxie Outcome-relevant war. Zusammengefasst konnten sie zwischen beiden Gruppen weder Unterschiede in den arteriellen Sauerstoffpartialdrücken bei Krankenhausaufnahme noch bei der Prognose feststellen. Die Daten ihrer Kohorte stellten sie unter der Creative Commons licence 4.0 anonymisiert und pseudonymisiert zur Verfügung [27]. Die Studie wurde damals von der Ethikkommission des Saint-Pierre-Universitätskrankenhaus und des Brugmann Universitätshospitals (Centre Hospitalier Universitaire Brugmann) genehmigt (beide Brüssel, Belgien, CE/17-05-07).

### Attributauswahl und Datenvorverarbeitung

Das öffentlich zugängliche Datenset enthält einzig Patienten mit ROSC. Selektiert wurden alle Datensätze, bei welchen der Beginn der Wiederbelebungsmaßnahmen (CPR, egal, ob Laienreanimation oder professionell) und der Zeitpunkt des ROSC dokumentiert war. Leider war die Erfassungsstruktur in der Datenquelle nicht vergleichbar zu den beispielsweise im Deutschen Reanimationsregister verwendeten Utstein-Kriterien [14]. Zudem mussten Attribute vermieden werden, welche auch nach dem ROSC dokumentiert worden sein könnten und daher zu einer Verzerrung geführt hätten. Als Attribute wurden daher lediglich Geschlecht, Alter, die Modified Rankin Scale vor CPR (mRS), der Ort des Herz-Kreislauf-Stillstands, beobachteter Herz-Kreislauf-Stillstand, Laienreanimation, Laienbeatmung, telefonische Reanimationsanleitung, defibrillierbarer Erstrhythmus und eine endotracheale Intubation vor ROSC ausgewählt. Daneben waren bereits die Dauer vom Herz-Kreislauf-Stillstand zum ROSC, CPR zum ROSC, Herz-Kreislauf-Stillstand bzw. CPR zur Erstdefibrillation, Herz-Kreislauf-Stillstand bzw. CPR zur ersten Medikamentengabe (ohne Angabe, ob Adrenalin oder Antiarrhythmikum, resp. Angaben zur Dosierung) verfügbar sowie die weiteren Zeitmarken Herz-Kreislauf-Stillstand bzw. CPR zu endotrachealer Intubation und Herz-Kreislauf-Stillstand bzw. CPR zu Anlage einer mechanischen Reanimationshilfe (hier Lund University Cardiopulmonary Assist System [LUCAS], Physio-Control/Jolife AB, Lund, Schweden). Das Attribut „erstes endtidales CO_2_“ wurde zur Modellierung nicht verwendet, obgleich es positiv mit der Qualität der Reanimationsmaßnahmen korreliert bzw. ein sprunghafter Anstieg auf einen ROSC hinweisend ist [25]. Da jedoch aus der Originalstudie dessen Dokumentationszeitpunkt (direkt nach Atemwegssicherung oder ROSC) nicht ermittelbar war, wurde dieser mögliche Confounder sicherheitshalber nicht verwendet. Ebenso war bei unbeobachtetem Herz-Kreislauf-Stillstand kein Ereigniszeitpunkt verfügbar, weswegen wir als Zielgröße für die Regressionsanalyse die Dauer vom Beginn der CPR zum ROSC auswählten. Fehlende Daten in den jeweiligen Fällen wurde nicht interpoliert, da die größtenteils kategorialen Attribute unabhängig voneinander waren und eine Interpolierung auch aufgrund des zu geringen Datensatzes als zu unsicher erschien.

### Modellierung und Algorithmus

Zur Regressionsanalyse wurde in der Software WEKA 3.9.6 (Waikato Environment for Knowledge Analysis, University of Waikato, Neuseeland, 2022) die Algorithmen M5P, modifizierte lineare Regression und ein Random Forest verwendet, welche alle Berechnungen mit einzelnen fehlenden Daten durchführen können.

Die multivariable lineare Regression (LR) berechnet einen linearen Zusammenhang zwischen Outcome und Prädiktoren anhand der Grundformel *y* *=* *a*_1_*x_*1*_ *+* *a*_n_*x_*n*_ *+* *…* *+* *b*. Dabei sind y die zu berechnende, abhängige Variable, x die unabhängige Variable, a der Regressionsparameter und b die stochastische Komponente (Achsenabschnitt). Vorteile sind ihre Einfachheit und Erklärbarkeit. Leider reagiert die LR sensibel auf Multikollinearität und kann leicht eine Über- oder Unteranpassung generieren. Zudem ist ihr Berechnungsverhalten restriktiv, da immer ein konstant linearer Zusammenhang postuliert wird. In vorliegender Studie wurde die LR mit der M5-Methode zur Attributsauswahl verwendet. Dieser untersucht für alle Regressionskoeffizienten das Akaike-Informationskriterium (AIC-Wert), welches ein Maß für die Güte der Anpassung als auch die Komplexität des Modells ist. Wenn sich der AIC-Wert durch Entfernen des Attributs mit dem kleinsten Regressionskoeffizienten verbessert, bleibt das Attribut entfernt; wenn der AIC-Wert sich verschlechtert, wird es wieder aufgenommen [3]. Als weitere Hyperparameter gemäß der Standardeinstellung („batch size“ = 100) wurden kollineare Attribute ausgelassen sowie die QR-Zerlegung und ein Ridge-Faktor von 1 ∙ 10^−8^ zur Reduktion von Überanpassung und zur Stabilitätserhöhung gewählt.

Beim M5P-Algorithmus wird dagegen ein Entscheidungsbaum erstellt, dessen Blätter lineare Regressionsgleichungen sind. Zunächst erfolgt eine Aufteilung des Datensatzes anhand der Minimierung der Varianz der Zielvariable. Anschließend wird eine multiple lineare Regression an einem Blatt durchgeführt. Auch hier wird die zuvor beschriebene M5-Methode zur Attributsauswahl in den Gleichungen genutzt. Vorteile sind eine schnelle und differenzierte Interpretierbarkeit. Nachteile entstehen auch hier bei nichtlinearen Berechnungen, durch die Empfindlichkeit gegenüber Ausreißern und durch die eingeschränkte Skalierbarkeit bei zu großen Datenmengen bzw. zu vielen Attributen [3, 31]. Als weitere Hyperparameter gemäß der Standardeinstellung („batch size“ = 100) wurde der Baum getrimmt sowie eine Glättung in der Berechnung durchgeführt („smoothing“), um Überanpassung und Extremwerte zu vermeiden.

Da die zuletzt genannten Nachteile durch moderne Ensemble-Learning-Methoden begrenzt werden können, wurde zudem der Random-Forest (RF)-Algorithmus verwendet. Bei RF wird eine bestimmte Anzahl an Zufallsbäumen (hier *n* = 100) erstellt, die jeweils eine Vorhersage treffen. Jeder Baum wird nur an einem zufälligen Teil der Trainingsdaten trainiert. Nicht alle Attribute werden in allen Bäumen berücksichtigt. Diese Vielfalt ergibt schlussendlich verschiedene Regressionswerte, welche vom Modell gemittelt werden. Vorteile von RF sind seine Robustheit gegen Überanpassung und die Modellierung von nichtlinearen Beziehungen. Er eignet sich zudem für große Datenmengen mit vielen Attributen, ist aber langsamer in der Berechnung und weniger gut interpretierbar [31]. Mehr Bäume stabilisieren die Vorhersagestabilität, da das Rauschen einzelner Bäume unterdrückt wird – jedoch ändert sich der Bias kaum, da er durch die Baumstruktur bestimmt wird. Mehr Bäume erhöhen zudem den Rechenaufwand und reduzieren das Overfitting nicht, weswegen die hier verwendete Baumzahl einen Kompromiss aus Vorhersagestabilität und Rechenaufwand darstellt. Als weitere Hyperparameter gemäß der Standardeinstellung („batch size“ = 100) wurden bei gleichwertigen Attributen jeweils eine Zufallsauswahl zur Baumaufteilung und eine maximale Tiefe ohne Begrenzung gewählt.

### Training, Testung und Vergleich

Alle Algorithmen wurden am Datensatz mittels 10facher Kreuzvalidierung 10-mal trainiert und getestet. Bei der 10fachen Kreuzvalidierung wird der Datensatz in 10 zufällige, gleich große Abschnitte unterteilt. Dabei dienen 9 Abschnitte zum Training und einer zur Testung. Das Verfahren wird so oft wiederholt, bis jeder Abschnitt einmalig zur Testung genutzt wurde. Anschließend werden die Ergebnisse gemittelt. Aufgrund der geringen Stichprobengröße wurde eine wiederholte 10fache Kreuzvalidierung verwendet, um die Varianz der Modellleistungsschätzungen zu reduzieren. Dabei führt eine Aufteilung in mehr Abschnitte gerade in kleinen Datensätzen theoretisch zu geringeren systematischen Fehlern (Bias), jedoch erhöht sich auch die Varianz der Testergebnisse, bei weniger Abschnitten verhält es sich entsprechend umgekehrt. Eine 10fache Kreuzvalidierung, welche 10-mal mit jeweils zufälligen Aufteilungspunkten wiederholt wird, erschien uns daher gegenüber einer 5fachen oder 15fachen als geeigneter Kompromiss hinsichtlich des Verhältnisses von Bias und Varianz. Gerade Letztere wird durch die 10fache Testwiederholung der Validierung noch besser gemittelt. Als Gütekriterien wurden der Pearson-Korrelationskoeffizient (KK) resp. das Bestimmtheitsmaß R^2^, der mittlere absolute Fehler („mean absolute error“, MAE), die Wurzel der mittleren Fehlerquadratsumme („root mean square error“, RMSE), der relative absolute Fehler („relative absolute error“, RAE) sowie der relative quadratische Wurzelfehler („relative root square error“, RRSE) verwendet. Der MAE berechnet sich aus der Summe der jeweiligen Fehlerbeträge (d. h. immer positiv), dividiert durch die Anzahl der Datenpunkte. Er wird kaum durch Ausreißer beeinflusst und ist leicht interpretierbar. Da analog zu den zeitlichen ROSC-Wahrscheinlichkeiten ca. nach Minute 30 auftretende Ausreißer erwartet wurden, mussten auch Letztere in der Fehlerdarstellung mitaufgenommen werden [8, 26]. Hierfür wurde der RMSE verwendet. Für seine Berechnung werden alle quadrierten, absoluten Fehler aufsummiert, durch die Anzahl an Datenpunkten dividiert und anschließend die Quadratwurzel gezogen. Die Quadrierung verstärkt die Wirkung eines Ausreißers stark (absoluter Fehler 2 min > 2^2^ = 4 vs. 3 min > 3^2^ = 9). Dadurch wäre der in der klinischen Anwendung zu erwartende Vorhersagefehler aber auch realistischer einschätzbarer. RAE und RRSE sind als Prozentangaben (0–100 %) des MAE bzw. RMSE (Einheit Minuten von 0 bis + ∞) zu verstehen. Höhere Werte für den KK und R^2^ sowie niedrigere Werte für RMSE, MAE, RAE und RRSE deuten auf eine bessere Effizienz der Modelle hin. KK können zwischen −1 und 1 liegen (negative bzw. positive Korrelation). Allgemein wird hierfür ein Wert zwischen 0,4 und 0,59 als moderat, 0,6 und 0,79 als stark und > 0,8 als sehr stark korrelierend angesehen. R^2^ dagegen liegt zwischen 0 und 1, wobei für R^2^ ein Bereich < 0,5 als schlechte Anpassung, 0,51–0,7 als akzeptable Anpassung, 0,71–0,9 als gute Anpassung und > 0,9 als exzellente Anpassung interpretiert wird [4, 12, 31]. Zudem wurde als weiteres Gütekriterium berechnet, bei wie vielen Fällen ein tatsächlicher ROSC erst nach dem Zeitintervall „berechneter ROSC + RMSE“ eingesetzt hätte – also eine Reanimation bei absolutem Vertrauen auf den Algorithmus vorzeitig abgebrochen worden wäre. Der statistische Vergleich der Gütekriterien zwischen den verschiedenen Algorithmen erfolgte mittels t‑ oder Exaktem Fisher-Test (*p* < 0,05 als signifikant, gewertet als hypothesengenerierender, explorativer *p*-Wert) in Microsoft Excel 2022 (Microsoft Corporation, Redmond, WA, USA). Zudem wurden der Mittelwert sowie die Varianz der tatsächlichen und berechneten Zeit von CPR bis ROSC auf Gleichheit mittels t‑ und F‑Test verglichen (*p* < 0,05 als signifikant, ebenfalls explorativ).

## Ergebnisse

### Datensatz

**Abb. 1:**
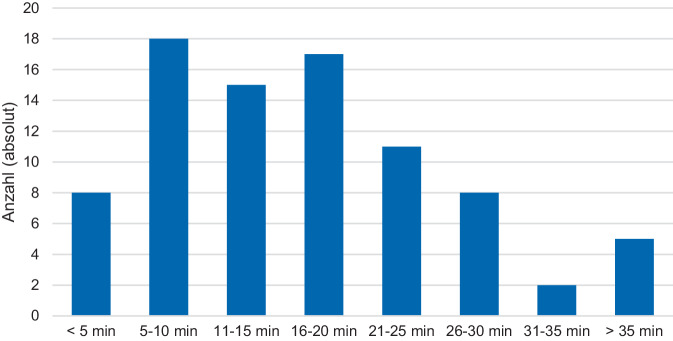
Verteilung der Dauer bis zum ROSC nach Beginn der Wiederbelebungsmaßnahmen; *ROSC* wiedereinsetzender Spontankreislauf, *min* Minuten

Es konnten von 92 Originaldatensätzen insgesamt 84 Datensätze (91 %) mit dem Zeitintervall Beginn CPR bis ROSC selektiert werden (16,83 min ± 9,78, mittleres Alter 65,7 Jahre ± 16,5, männliches Geschlecht 61,9 %). Bei 7 (8 %) lag der Zeitpunkt des Beginns der CPR bzw. bei einem (1 %) der Zeitpunkt des ROSC-Eintritts nicht vor, sodass keine Berechnung der Zeitdauer für die Regressionsanalyse herangezogen werden konnte. Es lag in 27,7 % ein defibrillierbarer Rhythmus vor. Die Quote der Laienreanimation betrug 48,1 % mit einer mittleren Dauer vom Herz-Kreislauf-Stillstand zu Reanimationsmaßnahmen von 5,13 min ± 6,03. Die mittlere Zeit von CPR bis zur ersten Defibrillation betrug 7,81 min ± 5,81 bzw. von CPR bis zur ersten Medikamentengabe 11,31 ± 7,23 min (für weitere Informationen: Tab. 1 und Abb. 1). Bei Krankenhausentlassung betrug der mediane mRS 6 (Interquartilsabstand 2,25–6), wobei ein mRS von 0–3 im Sinne eines guten neurologischen Outcome in 26,74 % vorlag (mRS 4–6 73,26 %). Das 30-Tage-Überleben wurde mit 34,78 % angegeben. Weitere Daten zur Häufigkeit von Hypothermiebehandlungen oder Koronarangiographien lagen nicht vor.

**Tab. 1:** Kenngrößen der Kohorte (*n* = 84)

Wert		Fehlende Werte
*Männliches Geschlecht*	61,9 %	*n* = 0
*Alter (Jahre)*	65,7 ± 16,52	*n* = 0
*mRS vor Herz-Kreislauf-Stillstand*	0 = 50 %	*n* = 8
	1 = 11,8 %	
	2 = 11,8 %	
	3 = 11,8 %	
	4 = 11,8 %	
	5 = 2,4 %	
*Ort des Herz-Kreislauf-Stillstands*	Öffentlich = 25,6 %	*n* = 2
	Zuhause = 59,8 %	
	Arbeit = 2,4 %	
	Pflegeheim = 12,2 %	
*Beobachteter Herz-Kreislauf-Stillstand*	83,3 %	*n* = 0
*Laienreanimation*	48,2 %	*n* = 1
*Laienbeatmung*	11,9 %	*n* = 0
*Telefonreanimation*	10,7 %	*n* = 0
*Defibrillierbarer Rhythmus*	27,7 %	*n* = 1
*Endotracheale Intubation vor ROSC*	58,3 %	*n* = 0
*Mechanische Reanimationshilfe*	22,9 %	*n* = 1
*Herz-Kreislauf-Stillstand bis CPR*	5,13 min ± 6,03	*n* = 13
*CPR bis 1. Defibrillation*	7,81 min ± 5,81	*n* = 47
*CPR bis Erstmedikament*	11,31 min ± 7,23	*n* = 17
*CPR bis mechanische Reanimationshilfe*	10,35 min ± 19,5	*n* = 68
*CPR bis ROSC*	16,83 min ± 9,78	*n* = 0

Sofern zutreffen Daten als Mittelwert mit Standardabweichung

*CPR* kardiopulmonale Widerbelebungsmaßnahmen, *ROSC* wiedereinsetzender Spontankreislauf, *mRS* modifizierte Rankin-Skala

**Abb. 2:**
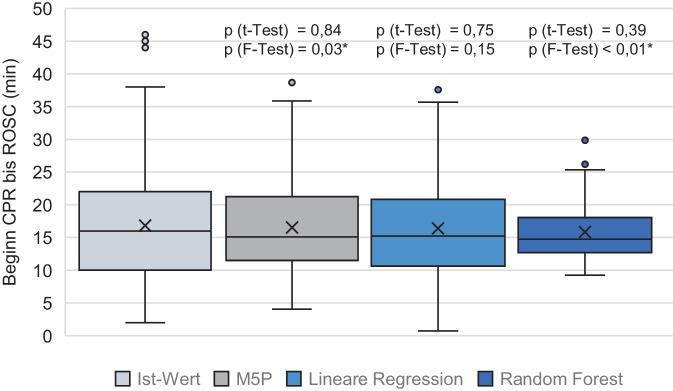
Box-and-Whisker-Plots zu den verschiedenen Algorithmen (Sollwerte) sowie zum Istwert. Lediglich bei der linearen Regression waren errechneter Mittelwert und Varianz nicht unterschiedlich zu den Istwerten; *CPR* kardiopulmonale Widerbelebungsmaßnahmen, *ROSC* wiedereinsetzender Spontankreislauf, *signifikant *p* < 0,05 im Sinne eines explorativen *p*-Werts

Mit M5P wurde im Durchschnitt eine Zeit von 16,55 min ± 7,70 zwischen CPR and ROSC vorhergesagt (t-Test *p* = 0,84 und F‑Test *p* = 0,03, Tab. 2). Der KK betrug 0,72 [95 %-Konfidenzintervall 0,70–0,73] (R^2^ = 0,52 [95 %-KI 0,50–0,53]) bei einem MAE von 5,27 min ± 0,25 [95 %-KI 5,11–5,43] bzw. einem RMSE von 6,84 min ± 0,24 [95 %-KI 6,69–6,99] (Abb. 2 und 3). Der Baum mit den Gleichungen ist in Abb. 4 dargestellt. Mit M5P kam es bei 11 Patienten zu einer ROSC-Zeitpunkt-Vorhersage, die vor dem tatsächlich ROSC-Zeitpunkt, inklusive dem RMSE, lag (13 %).

**Tab. 2:** Ergebnisse der Regressionsanalysen

	M5P	LR	RF	M5P vs. LR (*p*)	M5P/LR vs. RF (*p*)^(1)^
Korrelationskoeffizient	0,72 ± 0,02 [0,70–0,73]	0,73 ± 0,02 [0,72–0,74]	0,62 ± 0,02 [0,61–0,63]	0,23	< 0,01*
R^2^	0,52 ± 0,03 [0,50–0,53]	0,53 ± 0,03 [0,52–0,55]	0,38 ± 0,02 [0,37–0,40]	0,24	< 0,01*
MAE (min)	5,27 ± 0,25 [5,11–5,43]	5,18 ± 0,22 [5,05–5,32]	6,02 ± 0,08 [5,97–6,07]	0,45	< 0,01*
RMSE (min)	6,84 ± 0,24 [6,69–6,99]	6,76 ± 0,22 [6,63–6,9]	7,89 ± 0,11 [7,82–7,96]	0,51	< 0,01*
RAE (%)	67,58 ± 3,15 [65,62–69,53]	66,46 ± 2,99 [64,61–68,32]	77,21 ± 1,24 [76,44–77,98]	0,45	< 0,01*
RRSE (%)	69,64 ± 2,31 [68,21–71,07]	68,9 ± 2,39 [67,42–70,38]	80,36 ± 1,2 [79,61–81,10]	0,51	< 0,01*

*R*^*2*^ Bestimmtheitsmaß, *MAE* mittlerer absoluter Fehler („mean absolute error“), *RMSE* Wurzel der mittleren Fehlerquadratsumme („root mean square error“), RAE relativer absoluter Fehler („relative absolute error“), *RRSE* relativer quadratischer Wurzelfehler („relative root square error“), *min* Minuten, Angaben mit Standardabweichung und 95 %-Konfidenzintervall in eckigen Klammern

*Statistisch signifikant mit *p* < 0,05 im *Sinne* eines explorativen *p*-Werts

^(1)^Die sich nicht unterscheidenden Ergebnisse wurde für die jeweils getrennten t‑Tests M5P vs. RF und LR vs. RF zusammengefasst

**Abb. 3:**
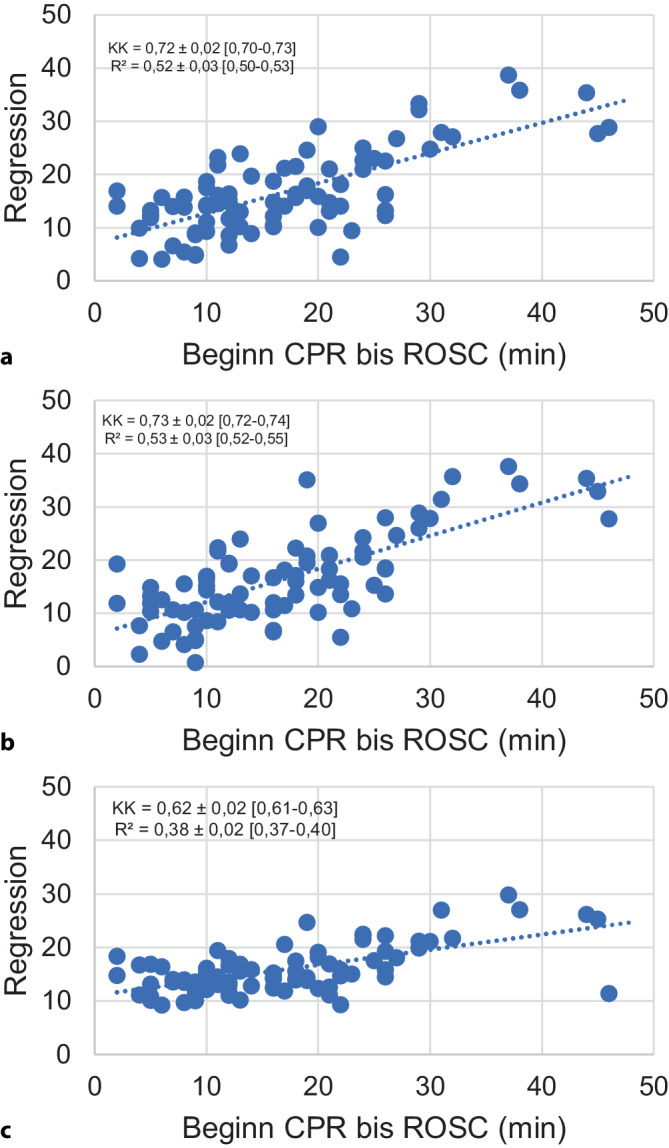
Diagramme mit tatsächlicher vs. berechneter Dauer bis zum Wiedereinsetzen des Spontankreislaufs für M5P (**a**), lineare Regression (**b**) und Random Forest (**c**). *CPR* kardiopulmonale Wiederbelebungsmaßnahmen, *ROSC* wiedereinsetzender Spontankreislauf, *KK* Korrelationskoeffizient, *R*^*2*^ Bestimmtheitsmaß, Angaben mit Standardabweichung und 95 %-Konfidenzintervall

**Abb. 4:**
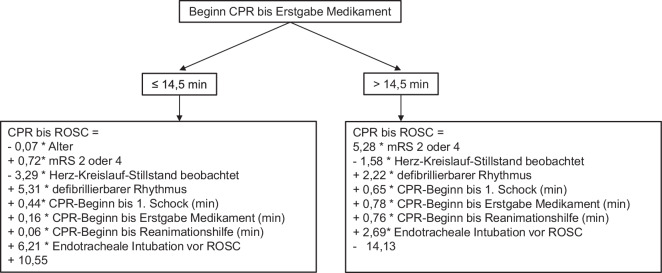
M5P-Baummodell mit linearen Regressionsgleichungen als Blätter; *CPR* kardiopulmonale Widerbelebungsmaßnahmen, *ROSC* wiedereinsetzender Spontankreislauf, *mRS* modifizierte Rankin-Skala vor Reanimation mit Wert 2 oder 4

Die Berechnung der modifizierten linearen Regression ergab folgende Formel:

$$\begin{aligned}&\textit{Zeit CPR-ROSC}=2{,}42*\\&(\textit{m\"{a}nnliches Geschlecht})+2{,}23*\\&(\textit{Ort Zuhause oder Pflegeheim})-3{,}81*\\&(\textit{Telefonreanimation}=\textit{ja})+3{,}73*\\&(\textit{defibrillierbarer Rhythmus}=\textit{ja})+\\&0{,}71*(\textit{Minuten von CPR-Beginn}\\&\textit{bis Schock})+0{,}81*(\textit{Minuten von}\\&\textit{CPR-Beginn bis Medikamentengabe})\\&+0{,}34*(\textit{Minuten von CPR-}\\&\textit{Beginn bis mechanische}\\&\textit{Reanimationshilfe})+4{,}24*\\&(\textit{Endotracheale Intubation vor ROSC}\\&=\textit{ja})+6{,}8*(\textit{mechanische}\\&\,\textit{Reanimationshilfe}=1)-12{,}25\end{aligned}$$

So wurden im Mittel 16,38 min ± 8,31 vom Beginn der CPR bis ROSC vorhergesagt (t-Test *p* = 0,75 und F‑Test *p* = 0,15). Der KK betrug 0,73 ± 0,02 [95 %-KI 0,72–0,74] (R^2^ = 0,53 [95 %-KI 0,52–0,55]) bei einem MAE von 5,18 min ± 0,22 [95 %-KI 5,05–5,32] bzw. einem RMSE von 6,76 min ± 0,22 [95 %-KI 6,63–6,9]. Der vorhergesagte ROSC-Zeitpunkt lag in 18 % vor dem tatsächlich beobachteten.

Random Forest berechnete eine Zeit von CPR bis ROSC von 15,83 min ± 4,45 (t-Test *p* = 0,39 und F‑Test *p* < 0,01). Der KK betrug 0,62 ± 0,02 [95 %-KI 0,61–0,63] (R^2^ = 0,38 [95 %-KI 0,37–0,4]) bei einem MAE von 6,02 min ± 0,08 [95 %-KI 5,97–6,07] bzw. einem RMSE von 7,89 min ± 0,11 [95 %-KI 7,82–7,96]. In 17 % wurde der ROSC-Zeitpunkt vorzeitig vorhergesagt. Die Unterschiede in dieser Vorhersage waren zwischen den Algorithmen nicht signifikant (Fishers Exakter Test alle *p* > 0,4). Allerdings waren die Unterschiede in KK, MAE und RMSE zwischen RF und M5P bzw. LR alle hochsignifikant (*p* < 0,01, kein signifikanter Unterschied zwischen M5P und LR, alle *p* > 0,2).

## Diskussion

Die vorliegende Studie evaluierte auf Basis eines vereinfachten Reanimationsdatensatzes die Schätzung der Dauer vom Beginn der CPR-Maßnahmen bis zum ROSC mittels ML-Regressionsanalysen. Die Algorithmen M5P bzw. die modifizierte lineare Regression zeigten eine starke Korrelation (KK > 0,7) bei noch akzeptabler Anpassung (R^2^ > 0,5). Bezüglich der Primärhypothese eines RMSE < 6 min (3 Reanimationszyklen oder 2 Adrenalingaben) verfehlte das beste Modell (LR: RMSE 6,76 min) diese knapp. Ursächlich hierfür erscheinen Limitationen in der Kohortengröße und Datenstruktur. Bezogen auf die Sekundärhypothese konnte einzig LR für die Gesamtkohorte sowohl den Mittelwert als auch die Varianz ohne signifikante statistische Abweichung von der tatsächlichen Zeitdauer schätzen. Sofern einzig auf den errechneten Wert plus RMSE vertraut worden wäre, wären die Reanimationsbemühungen in ca. einem Sechstel der Fälle vor Einsetzen eines ROSC beendet worden. Die dargelegten Modellgüten wären für die Individualmedizin derzeit noch zu ungenau und würden den gestellten Anforderungen an Regressionsmodelle in solch hochkritischen Situationen bei Weitem nicht genügen. Die Studie ist nach Wissen der Autoren die erste, welche den ROSC-Zeitpunkt direkt berechnet. Die vielversprechenden Studienergebnisse legen aber eine Machbarkeit eines Regressionsmodells nahe, wovon weitere Schritte in der zukünftigen Entwicklung abgeleitet werden können [20, 30].

### Datenbasis

Die Studie wurde im Sinne einer ersten Machbarkeitsstudie an frei verfügbaren, allerdings vereinfacht erfassten Studiendaten an einer kleinen Kohorte in einem dem deutschen vergleichbaren Rettungssystem durchgeführt. In Belgien kommen neben Notärzten und Notarzteinsatzfahrzeugen auch examinierte Notfallkrankenpfleger als Notfallsanitäter zum Einsatz [22]. Ein Bias ergibt sich bereits aus der urbanen Studienumgebung mit universitärer Krankenhausversorgung. Auch die Zielsetzung der ursprünglichen Studie war nicht vergleichbar mit der hier untersuchten Fragestellung. Die von Malinverni et al. gewählte Datenstruktur war simpler und entsprach nicht den im Deutschen Reanimationsregister verwendeten Utstein-Kriterien [14, 23]. So fehlen beispielsweise Daten zur Anzahl der Defibrillationsversuche und Adrenalingaben, zur Gabe von Antiarrhythmika, Zeitpunkt und Art der ersten Beatmung, zum erweiterten Atemwegsmanagement sowie zum Medikamentenzugang. Auch Angaben zu den Anfahrtszeiten, Ätiologie, potenziell reversiblen Reanimationsursachen bzw. zu deren Diagnostik (z. B. Ultraschall) oder Therapie (z. B. Thoraxdrainage) waren nicht vorhanden. Daten zu Patienten ohne ROSC lagen nicht vor. In Bezug auf Patientengeschlecht, Ort des Herz-Kreislauf-Stillstands, Alter, defibrillierbarem Rhythmus und Laienreanimationsquote wiesen die vorliegenden Daten sowie die Angaben des Jahresberichts 2023 des Deutschen Reanimationsregisters ähnliche Werte auf [11]. Bei der Quote der beobachteten Herz-Kreislauf-Stillstände (Deutschland 42,4 % vs. 83 %) und der Telefonreanimationen (Deutschland 33,9 % vs. 10 %) ergaben sich jedoch Unterschiede, die durch diesen Selektionsbias erklärbar wären. Nach Angabe des Reanimationsregisters lag die Verwendungshäufigkeit von mechanischen Reanimationshilfen um die Hälfte niedriger (10,4 % vs. Studie 22,9 %). Zwar zeigt sich in Metaanalysen eine gewisse Evidenz für eine vermehrte ROSC-Rate mithilfe dieser Geräte, welche sich allerdings nicht in einem besseren Outcome niederschlägt [7]. Daher werden sie von der Leitlinie nicht generell, sondern v. a. situativ z. B. für den Transport unter Reanimation empfohlen [25]. Die No-Flow-Zeit war in vorliegenden Daten gut 1 min kürzer als im Reanimationsregister (6,12 min). Weitere Zeitmarken waren nur eingeschränkt vergleichbar, da im Register die Zeiträume vom Einsatzalarm ausgehend veröffentlicht werden. Bezogen auf das 30-Tage-Überleben wurde im Register eine Quote von 25,08 % angegeben, welche in der Studienpopulation um den Faktor 1,38 höher lag (34,78 %) [11]. Ob dies dem Studienaufbau, einer Standortselektion oder einer qualitativ höheren Versorgung geschuldet ist, kann abschließend aus den vorliegenden Daten nicht festgestellt werden. Die EuReCa-TWO-Studie ermittelte allerdings für das Krankenhausüberleben eine Rate von 29,7 % (Deutschland) bzw. 18,7 % (Belgien) bei vergleichbarer durchschnittlicher ROSC-Rate (42 %) bzw. Laienreanimationsquote (46 %) [14]. Da das Deutsche Reanimationsregister insgesamt eine erweiterte, genauere Datenstruktur und Studiengruppe darstellt, welche internationalen Standards in der Reanimatologie entspricht, würde es sich für die weitere Modellentwicklung gerade mit Blick auf externe Validierungen eignen.

### Modellentwicklung

Prinzipiell sind zwei Modellentwicklungen, basierend auf einem Regressionsmodell, vorstellbar. Eine rein klinische Anwendung könnte Notärzte während der Reanimation direkt am Einsatzort unterstützen. Unter der Prämisse einer möglichst genauen Regressionsvorhersage könnten Notärzte entweder frühzeitig bei der Planung der Einsatztaktik unterstützt werden, z. B. frühzeitiger Transport ggf. sogar unter Reanimation, Alarmierung von weiteren Rettungskräften (z. B. Feuerwehr bei aufwendiger technischer Rettung), nahtlose klinische Versorgung (z. B. Aktivierung eines Herzkatheterteams) oder aber realistische Prognoseabschätzung („resuscitation time bias“). Doch welche Zielwerte wären für mit Blick auf die Anforderungen an solch ein Modell praktikabel, etwa ein RMSE von ca. 4 min (2 Zyklen oder eine Adrenalingabe)? Dies würde auch eine Gefahr der Überanpassung des Modells in sich bergen und könnte zu vorzeitigen Therapieabbrüchen führen. Ungeklärt ist auch, wie die Darstellung der Ergebnisse – als Endpunkt oder als Zeitfenster – den Entscheidungsprozess während der Reanimation beeinflussen würde [20]. Unabhängig davon wären für den Erfolg solch eines Modells aber die zeitliche Auflösung und zeitnahe Dokumentation des Reanimationsablaufs zusammen mit einer dynamischen Berechnungsanpassung entscheidend. Zweitens wäre zur effektiven Anwendung des Modells eine Verknüpfung mit einem Programm zur generellen Vorhersage eines ROSC notwendig. Aktuelle Modellierungen hierzu weisen zwar eine ausreichende Klassendiskrimination auf, sind aber bezogen auf äquivalent hohe Sensitivität, Spezifität und positiv/negativ prädiktive Werte noch nicht ausreichend robust [2, 8, 18]. Zudem stammen diese Berechnungen mehrheitlich aus dem koreanischen Rettungsdienstsystem, in dem Paramedics ohne ärztliche (Tele‑)Supervision eine Reanimation nur bei sicheren Todeszeichen beenden resp. selbstständig erweiterte Reanimationsmaßnahmen wie Adrenalingaben nicht immer durchführen dürfen. Daher thematisierten diese Untersuchung v. a. die optimale Dauer der Reanimationsmaßnahmen vor Ort unter dem Gesichtspunkt einer „Stay-and-Play“- vs. „Scoop-and-Run“-Versorgungsstrategie [8, 24]. Park et al. evaluierten für eine Gesamtkohorte verschiedene Regressionsmodelle zur ROSC-Wahrscheinlichkeit und integrierten dann darin eine empirische, linear abnehmende Überlebenswahrscheinlichkeit im Sinne einer zeitlichen ROSC-Vorherhersage. Dies führte zu einer verbesserten Robustheit und Kalibrierung der Modelle [24]. Choi et al. modellierten dagegen eine hochindividualisierte Versorgungsstrategie. Neben einer potenziellen Erhöhung der Überlebensrate von 9,6 % auf 12,5 % ergab sich eine bimodale empfohlene maximale Reanimationszeit vor Ort vor Weitertransport unter Reanimation – mit kurzen Zeiten (< 6 min) für jüngere Patienten mit schockbarem Rhythmus und bei Verfügbarkeit von extrakorporaler klinischer Reanimation sowie längeren Zeiten (28 min) für Patienten mit ungünstigen Ausgangsbedingungen wie Asystolie [8]. Beide Studien integrierten zwar eine zeitabhängige ROSC-Wahrscheinlichkeit, lassen sich aber wegen der unterschiedlichen Rettungssysteme nicht auf hiesige Verhältnisse übertragen. Zudem wäre die Frage der konsequenten Umsetzung solch einer Therapiestrategie in Anbetracht knapper klinischer Ressourcen hierzulande ungeklärt.

Eine zweite Einsatzmöglichkeit bietet sich im Qualitätsmanagement aufgrund der Nichtunterschiedlichkeit von Mittelwert und Varianz der Ist- und Sollwerte des LR-Modells an. Ähnlich dem RACA-Score könnte solch ein Modell in Registern helfen, die tatsächliche zur vorhergesagten Dauer bis zum ROSC zu analysieren, um Leistungsfähigkeit und Effizienz der Reanimationsmaßnahmen für eine Gesamtkohorte oder Rettungsdienstbereiche zu bewerten [13]. Neben einer zeitnahen Umsetzung könnte das Instrument zudem international validiert werden und würde zudem weitere Evidenz für ein späteres klinisches Programm generieren [19, 30]. Die Modellierung muss an einer qualitativ hochwertigen und großen Kohorte mit ROSC erfolgen, sollte dann aber im zweiten Schritt auch bei erfolgloser Reanimation untersucht werden, gerade wenn eine hohe A‑priori-Wahrscheinlich für einen ROSC vorgelegen hätte. Das Ziel wäre beispielsweise eine Überprüfung ausreichend langer Reanimationsmaßnahmen in dieser Kohorte. Für solch ein Modell würden sich analog zur Entwicklung des RACA-Scores Datensätze der Referenzstandorte des Deutschen Reanimationsregisters anbieten [13]. Neben Patientencharakteristika, Zeitmarken der Rettungskette, Umständen und Ursachen des Herz-Kreislauf-Stillstands, Laienreanimation sowie Zeitpunkte, Art und Weise und Ablauf des Atemwegsmanagements, der Defibrillation und der medikamentösen Therapie könnten auch Daten aus den Defibrillatoren bzw. Feedbacksystemen genutzt werden, sofern sie sicher vor dem ersten ROSC erfasst wurden. Auch die endtidalen CO_2_-Werte vor ROSC könnten dann in die Modellierung einfließen. Nach Training und Testung könnten ein nichtverwendetes Datenset aus der Referenzstandortgruppe und ein Datenset aus den übrigen Standorten (als weniger qualitativ hochwertige Echtweltdaten) zur internen Validierung genutzt werden.

### Limitationen

Der verfügbare Datensatz enthält von Grund auf nur Daten zu Patienten mit ROSC, sodass hier ein Selektionsbias möglich erscheint. Weitere Limitationen ergeben sich aus dem retrospektiven, monozentrischen Studiendesign in einer Metropole mit universitärer Krankenhausversorgung, der geringen Kohortengröße, welche zu einer stärkeren Gewichtung von Ausreißern (RMSE) führen kann, sowie den verwendeten Algorithmen. Zudem war der Anteil von teilweise fehlenden Daten erhöht, wie es bei Echtweltdaten zu erwarten ist. Strategien zur Interpolierung wurden aufgrund des zu geringen Datensatzes, der Echtweltdatenvergleichbarkeit bzw. der z. T. kategorialen Variablen verworfen, ebenso eine Aufteilung des Datensatzes in Training, Testung und Validierung. Die Regression fehlender Werte hätte zudem zu einem systematischen Bias führen können. Aufgrund des kleinen Datensatzes wurden außerdem zusätzliche Analysen mit einem künstlichen neuronalen Netzwerk verworfen, da ein ausreichend großer Trainingsdatensatz nicht generiert werden konnte [16]. Als Kompromiss wurde daher RF verwendet. Da die Beeinflussung durch Ausreißer in der verfügbaren Kohortengröße stärkere Gewichtung erfährt, bleibt abzuwarten, ob in einem größeren Datensatz bessere Resultate erzielt werden können. Außerdem muss geprüft werden, ob dann die auf linearer Regression basierenden Modelle Probleme mit Multikollinearität zeigen bzw. über- oder unterangepasst sind [3]. Da der veröffentlichte Datensatz nur Patienten mit ROSC enthält, konnten leider nicht mittels weiterer Analysemethoden wie beispielsweise der Cox-Regression signifikante Prädiktoren bzw. deren zeitlicher Einfluss ermittelt werden, weswegen zur Abschwächung dieser Limitation der M5P-Algorithmus verwendet wurde. Leider wurde in den verfügbaren Daten zudem nicht weiter auf die Qualität der Reanimationsmaßnahmen (wie z. B. adäquate Kompression), zur Ausgangssituation per se (z. B. Vorerkrankungen) sowie auf die potenzielle Ursache des Herz-Kreislauf-Stillstands eingegangen, sodass Untergruppen ggf. nicht ausreichend repräsentiert waren.

## Fazit für die Praxis

- Die Zeitdauer vom Beginn der Reanimationsmaßnahmen bis zum ROSC scheint sich regressiv mittels maschinellen Lernens abschätzen zu lassen.
- Allerdings bedarf es einer Ausweitung der Datengrundlagen, um verbesserte Prognosemodelle erstellen zu können.
- Diese könnten zunächst zur Qualitätssicherung in Registern genutzt werden, um dann, weiterentwickelt, auch klinisch während der Reanimation eingesetzt zu werden.

## Data Availability

Die in dieser Studie ermittelten Modelle können auf begründete Anfrage beim Korrespondenzautor angefordert werden. Der zugrundeliegende Datensatz ist unter der Creative Commons Licence 4.0 frei abrufbar [27].
